# Gold Plate Electrodes Functionalized by Multiwall Carbon Nanotube Film for Potentiometric Thallium(I) Detection

**DOI:** 10.3390/nano9081160

**Published:** 2019-08-14

**Authors:** Saad S. M. Hassan, Sabah. M. Abdelbasir, M. Abdelwahab Fathy, Abd El-Galil E. Amr, Mohamed A. Al-Omar, Ayman H. Kamel

**Affiliations:** 1 Chemistry Department, Faculty of Science, Ain Shams University, Abbasia 11566, Cairo, Egypt; 2 Electro Chemical Treatment Dept., Central Metallurgical Research and Development Institute (CMRDI), P.O. Box 87, Helwan 11421, Cairo, Egypt; 3 Pharmaceutical Chemistry Department, Drug Exploration & Development Chair (DEDC), College of Pharmacy, King Saud University, Riyadh 11451, Saudi Arabia; 4 Applied Organic Chemistry Department, National Research Center, Dokki 12622, Giza, Egypt

**Keywords:** solidcontact ISEs, thallium, ion-to-electron transducer, multiwall carbon nanotubes (MWCNTs)

## Abstract

Solid-contact potentiometric ion-selective electrodes (SC-ISEs) for thallium determination have been designed using multiwall carbon nanotubes (MWCNTs) as the ion-to-electron transducer. Dispersed MWCNTs were drop-casted over a gold plate electrode. Two different crown ethers were used in the sensing membrane for the recognition of thallium (I). Sensorsbased on dibenzo-18-crown-6 (DB18C6) as a neutral carrier and NaTPB as an anionic additive exhibited a near Nernstian response of 57.3 mV/decade towards Tl^+^ ions over the activity range 4.5 × 10^−6^–7.0 × 10^−4^ M, with a limit of detection of 3.2 × 10^−7^ M. The time required to achieve 95% of the steadyequilibrium potential was <10 s. The complex formation constant (log *β_ML_*) between dibenzo-18-crown-6 and thallium (I) (i.e., 5.99) was measured using the sandwich membrane technique. The potential response was pH independent over the range 3.0–9.5. The introduction of MWCNTs as an electron-ion-transducer layer between gold plate and the sensing membrane lead to a smaller membrane resistance and a large double layer capacitance, which was proven using impedance spectra and chronopotentiometry (i.e., 114.9 ± 12 kΩ, 52.1 ± 3.3 pF, 200 ± 13.2 kΩ, and 50 ± 4.2 µF). Additionally, reduction ofthe water layer between the sensing membrane and the underlying conductor wastested. Thus, it is clear that MWCNTs can be used as a transducing layer in SC-ISEs. The proposed sensor was introduced as an indicator electrode for potentiometric titration of single and ternary mixtures of I^−^, Br^−^, and S^2−^ anions.

## 1. Introduction

Natural processes and human activity increase the concentration of metals in the environment. This poses a threat to human and other living organisms. One of the most significantpollutants that isintroduced and affects the environment is thallium (Tl), which is produced as waste from lead and coal combustion [1]. Thallium is the most dangerous pollutant, with a higher toxicity to the biosphere than Cd, Cu, Hg, and Pb [2]. It is used in the manufacture of low-temperature thermometers, optical lenses, semiconductors, jewelry, and as a catalyst in certain alloys [3]. Thallium (I) sulfate is also used as an insecticide and for rat poison [4]. Soluble thallium (I) compounds, e.g., thallium nitrate, acetate, and carbonate are dangerous because absorption within the body by ingestion or skin contact leads to high toxicity [5]. Athalliumconcentration of 0.5 mg/100g of tissue can be regarded as thallium poisoning [5]. 

From a biological and environmental point of view, determination of thallium (I) ion concentration has become a major concern for researchers. There are several techniques that have been used to determine thallium (I) ion in solution, including differential pulse anodic stripping voltammetry (DPASV) [6,7], square wave anodic stripping voltammetry (SWASV) [8], flame atomic absorption spectrometry (FAAS) [9], emission spectrometry [10], inductively coupled plasma (ICP) [11,12], graphite furnace atomic absorption spectrometry (GFAAS) [13], and spectrophotometry [14]. Despite the sensitivity of these methods, they suffer from the need for expensive instrumentation. For this reason, the need for low-cost, simple, miniaturized, and accurate analytical techniques for Tl determination becomes a strong requirement. Ion-selective electrodes (ISEs) have been used extensively as potentiometric sensors in chemical analysis for several analytes [15,16]. Conventional or symmetrical ISEs, for which there is an inner filling solution that acts as a conducting electrolyte between the inner reference electrode and the ion selective membrane (ISM), suffer from drawbacks such aslack ofportability, largesize, and their need for routinemaintenance, thus limiting their wider applications [17,18]. 

On the other hand, solid-state ion-selective electrodes have abandoned the internal filling solution by coating the ISM directly onto the solid conducting substrates. Consequently, some of the drawbacks mentioned above have been eliminated [19]. The first example of solid-state ISEs was a coated-wire electrode (CWE), which was invented in the 1970s [20]. However, these types of electrode have the disadvantage of poor long-term potential stability. This can be attributed to the indefinite phase boundary potential at the interface between the metallic substrate and the sensing membrane. To solve this problem, an intermediate layer was used to act as an ion-to-electron transducer between the metallic substrate and the sensing membrane, and significant efforts have been made to investigate the effective transducer layers [21]. To date, many solid contact layers such as carbon, conducting polymers, and nobel metal materials have been reported [22,23,24]. 

However, these systems have some limitations, including limited selectivity, the need for calibration, and potential drift, so they require significant improvement. Certainly, it is clear that the potential stability is the most important aspect. There are two reasons for the potential drift: the water layer formation between the ISM and the solid substrate, and the non-zero current. In the first case, due to poor adhesion of the membrane to the substrate, the sensor is affected by osmolality variations, which leads to delamination during operation [20]. Nanomaterials have many advantages as a solid contact overconducting polymers, which suffer from the possibility of side-reactions, lower conductivity compared to the nanomaterials, and a sensitivity to light and pH [25]. 

In the systems containing nanomaterials, the interfacial potential is related to the amount of charge accumulated in the double layer, not to ion partitioning as in symmetrical ISEs, or to reduction-oxidation reactions as in the case of using conducting polymers. Nano-structured material prevents the risk of water absorption and enables the achievement of good adhesion because of their hydrophobic behavior and large surface area. Furthermore, they form large double layer capacitance values, which reduce the effect of polarization due to non-zero currents [26]. Nobel metals and carbon nanostructures have been investigated for use as solid-contacts for ISEs. Examples of these carbon nanostructures are porous carbon [27], carbon nanotubes [28,29,30,31], graphene [31,32,33], fullerene [34,35], and three-dimensionally ordered macroporous (3DOM) carbon [36,37]. These carbon-nanostructure materials are chemically stable and exhibit high specific surface areas. Examples of nobel metals are platinum nanopetals [38], gold nanoparticles [39,40], and gold nano-clusters [41]. Besides the solid- contact itself, the choice of electron conducting substrate is also important. The nature of the electron conductor substrate has an effect on the interface between this substrate and the solid-contact material. The equilibration times of all-solid-state ISEs using different electron-conducting substrates (i.e., glassy carbon, Au, and Pt) were tested [42]. Sensors using glassy carbon and Au as electron conducting substrates exhibited much shorter equilibration times than electrodes made from Pt substrate. Similarly, sensors made with Au substrates were also reported to exhibit higher *E°* reproducibility than those based on glassy carbon substrates [43].Therefore, careful consideration should be taken inthe choice of the electron-conducting substrate (i.e., glassy carbon, Au, and Pt) when designing solid-contact ISEs.

In this work, miniaturized solid-contact potentiometric ion-selective electrodes (SC-ISEs) for Tl^+^ determination are presented for the first time. The sensor membranes are based on the use of dibenzo-18-crown-6 (DB18C6) as a neutral carrier. Multiwall carbon nanotubes (MWCNTs) are used as a solid contact material on a gold film (i.e., electron-conducting substrate). The constructed Tl^+^-SC-ISEs exhibit high potential stability and small membrane impedance. This simple fabrication of the electrode design permits the fabrication of these ISEs by a non-specialist. The developed SC/Tl^+^-ISEs are fully characterized and tested in potentiometric titration of single and ternary mixtures of I^−^, Br^−^, and S^2−^ ions. As a consequence of the excellent analytical performance, we conclude that this technology may become promising for the design of in-situ Tl^+^ sensing probes.

## 2. Materials and Methods

### 2.1. Equipment

Potential measurements were performed with an Orion (Cambridge, MA, USA) Model 720/SA pH/mV meter at 25 ± 1 °C using the gold plate thallium polymeric membrane sensor in conjunction with Ag/AgCl double-junction reference electrode (Orion 90-20) filled with 1 M CH_3_COOLi. Chronopotentiometry and electrochemical impedance spectroscopy (EIS) measurements were carried out using a potentiostat/galvanostat (Autolab Model 2000, Metrohom Instruments, Herisau, Switzerland). A three-electrode configuration cell containing a silver/silver chloride (3 M KCl) reference electrode and an auxiliary electrode made from platinum wire was employed. The impedance spectra were measured and recorded at open-circuit potential in a 0.01 M NaClO_4_ solution with an excitation amplitude of 10 mV and a frequency range of 100 kHz–0.1 Hz.

### 2.2. Materials and Reagents

All chemicals and reagents used were of analytical grade and were used as received and prepared with de-ionized water. For membrane preparation, high molecular weight poly (vinyl chloride) (PVC), O-nitrophenyloctyl ether (o-NPOE), Tetrahydrofurane (THF), and sodium tetraphenylborate (NaTPB) were used as received from Fluka or Merck. Multiwall carbon nanotubes (MWCNTs) were purchased from (EPRI, cairo, Egypt). Sodium sulphide (Na_2_S), sodium iodide (NaI), sodium bromide (NaBr), and Trizma^®^ buffer were purchased from Sigma Aldrich. A 0.1 M stock solution of TlNO_3_ was freshly prepared. 

### 2.3. Preparation of Gold Plate Electrodes

Gold plate electrodes (GPEs) witha gold diameter of 4 mm and a purity of 99.999% were sonicated for 10 min in acetone then cleaned by isopropanol and ethanol, each for 10 min. De-ionized water was then used to rinse the electrodes for 5 min, which were then left for 2 hto dry. Commercial multiwalled carbon nanotubes (MWCNTs) with a diameter of 1–20 nm were sonicated for 30 min with tetrahydrofuran (THF) in order to get a good dispersion for the carbon nanotubes (CNTs) [44,45]. Using a micropipette, droplets of the CNTs solution with THF were dropped over the gold plate electrode. As a result, CNTs were uniformly deposited over the gold plate electrode. It was then left for 2 h for drying by evaporation of tetrahydrofuran from the electrode.

### 2.4. Sensor Construction

**Figure 1:**
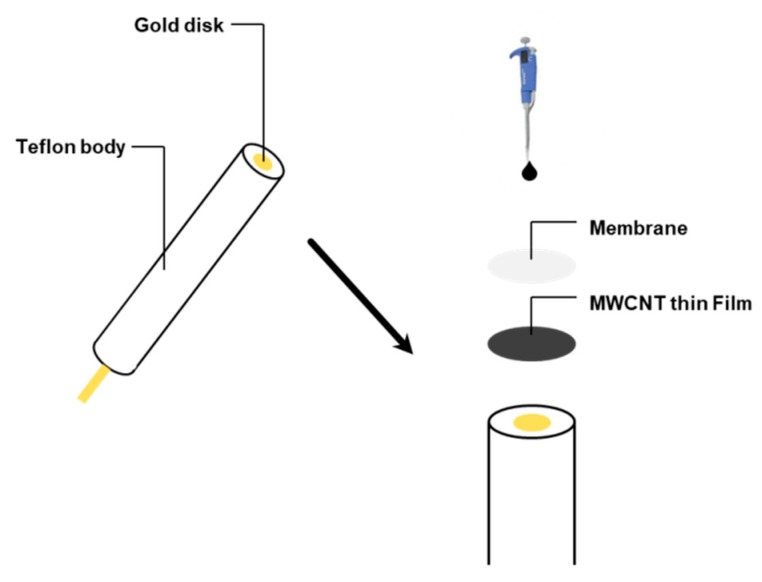
Sensor construction using multiwalled carbon nanotubes (MWCNTs)

The ion selective membranes were prepared by dissolving appropriate amounts of ionophores, NaTPB as an anionic additive, o-NPOE as a plasticizer, and PVC in THF (3 mL) as recommended bythe Internatioal Union of pure Applied chemistry (IUPAC) [46]. 50 μL of the homogenous membrane cocktail was drop-casted on the gold plate electrode directly, or on the CNTs deposited on the gold plate electrode, using a micropipette and then leftfor 2 h for drying by the evaporation of tetrahydrofuran as shown in Figure 1. Thereafter, the sensors were conditioned by soaking them in a 0.01M aqueous solution of TlNO_3_ for 12 h.

### 2.5. Potential Measurements

The proposed ISE was calibrated using a standard two electrode (working and reference) cell configuration, using a gold plate electrode and a CNT/gold plate electrode in conjunction with an Ag/AgCl reference electrode. The cell solution contentsof 10.0 mL of 0.01 M Trizma buffer (pH ~6) and (0.5–1.0) mL aliquots of a 1.0 × 10^‒1^–1.0 × 10^‒7^ M from an aqueous solution of Tl^+^ were sequentially added. After potential stabilization, the readings were plotted as a function of log a_Tl+_. Estimation of Tl^+^ concentration was done using the calibration plot.

### 2.6. Complex Formation Constants Measurements

According to the sandwich membrane method, experiments were carried out [47,48]. The PVC/plasticizer (1:2) membranes containing 80 mmol/kg of ionophore and 56 mmol/kg of NaTPB (total membrane mass 100 mg) were cast. Additionally, membranes without ionophore were prepared by the same constituents. A 50 μL of the membrane cocktail was drop-casted from both types of membrane and then conditioned overnight in a 0.01 M solution of the appropriate salt (Tl^+^, K^+^, Pb^2+^, Na^+^, NH_4_^+^). The membrane with the ionophore faced to the sample solution. The membranes were then instantly placed into a salt solution that was identical to the conditioning of the membrane. The mean potential was recorded for the last minute of a 10 min measurement period in the test solution. In a separate measurement, the potential of an electrode with the membrane containing no ionophore was measured in the same solution. The formation constant (log$\beta_{ML}$) was calculated according to Equation (1) [47,48]:

(1) $$log\;\beta_{ML}=\frac{E_{M}\cdot z_{I}F}{2.303\cdot RT}-n\;log(L_{T}-\;\frac{n}{z_{I}}R_{T}),$$

where *n* is the complex stoichiometry, *L_T_* is the total concentration of ionophore, *R_T_* is the concentration of the lipophilic ionic site additives, and *R*, *T*, and *F* are the gas constant, the absolute temperature, and the Faraday constant, *Z_I_* charge of the tested ion. The membrane potential *E_M_* is determined by subtracting the cell potential for the membrane without ionophore from that of the sandwich membrane.

## 3. Results and Discussions

### 3.1. Performace Characterisics of the Proposed Sensors

**Figure 2:**
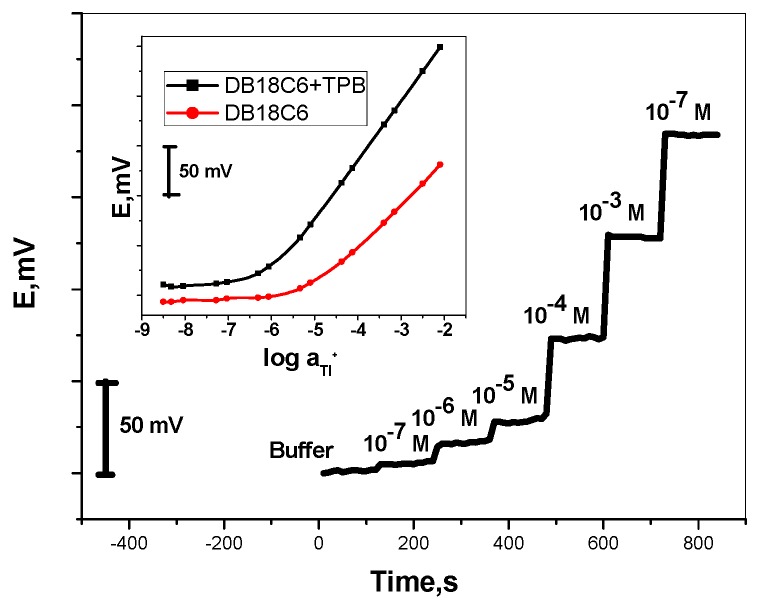
Time trace of the proposed sensors. Insert: Calibration curves of these ion sensors.

Attempts were made to use two different crown ethers as neutral carriers for the Tl^+^ ion in the PVC matrix membrane sensors. For ISEs based on neutral carriers, the potentiometric selectivity depends mainly on the ability of the neutral carrier to extract the desired ion [49,50]. According to the previously reported studies for solvent extraction [51], some crown ether derivatives revealed good complexing ability towards the Tl^+^ ion. In this work, dibenzo-18-crown-6 (DB18C6) was tested as a neutral carriers for designing a miniaturized SC/Tl^+^-ISEs. MWCNTs were used as an intermediate layer between the sensing membrane and Au, which acts as an electron conductor substrate. The membrane composition was 32.0 wt.% PVC, 65.0 wt.% plasticizer, and 3.0 wt.% ionophore. Potentiometric characteristics of sensors based on DB18C6 revealed a strong response towards Tl^+^ ions. Results from replicate studies showed sub-Nernstian slopes of 32.7 ± 2.5 mV/decade, with detection limits of 4.0 × 10^−6^ M, respectively. The addition of 1.5 wt.% Na-TPB enhanced the sub-Nernstian calibration slope from 32.7 ± 2.5 to a Nernstian slope of 57.3 ± 1.6 mV/decade and lowered the detection limit from 4.0 × 10^−6^ M to 3.2 × 10^−7^ M. The addition of NaTPB as lipophilic anions to the membrane improved its performance characteristics, because it stabilizes membrane operating conditions, decreases membrane resistance, and significantly reduces response times. The calibration plots of these ISEs are shown in Figure 2, and their performance characteristics are presented in Table 1. The potential responses revealed by the proposed sensors can be attributed to the selective interaction of Tl^+^ ions with DB18C6 and are affected by: (i) The ionic diameter of the crown ionophore; (ii) the ionic size of the thallium (I) ion; and (iii) the spatial configuration of this ionophore. The ionic size of Tl^+^ is 1.73 Å, while the ionic diameters of both 18-crown-6 varies from 2.6–3.2 Å [52]. 

The response time of the sensor was also tested by measuring the time required to achieve a 95% steady potential for all concentration ranges (10^−6^–10^−2^ M TlNO_3_ solutions). Fairly short response times of 5 s for [Tl^+^] > 10^−5^ M and 10 s for [Tl^+^] ≤ 10^−5^ M were obtained (Figure 2). A drop in the sensitivity was observed after 1 week of reasonably stable behavior. 

**Figure 3:**
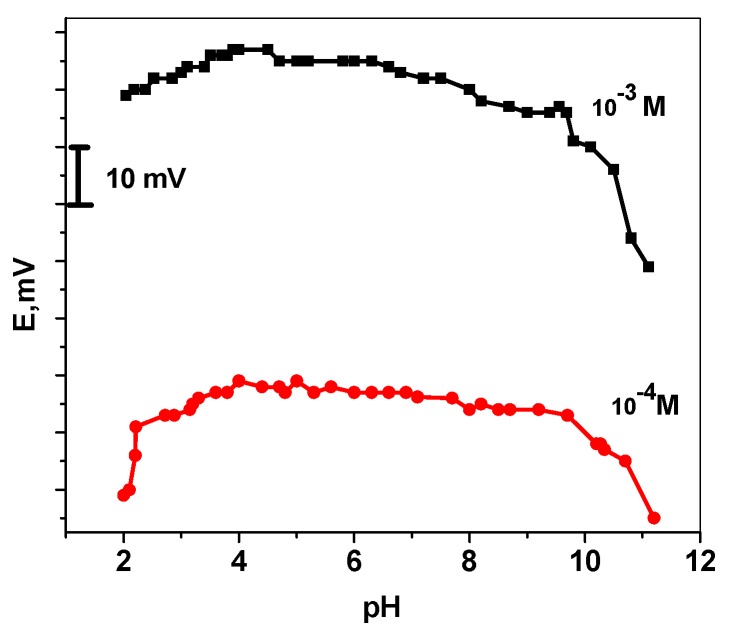
Effect of pH on the response of the sensor.

The pH dependence of the DB18C6+TPB membrane based sensor was tested using two Tl^+^ concentrations (i.e., 10^−3^ and 10^−4^ M). A stable potential response was noticed over the pH range 3–9.5, as shown in Figure 3. A pH adjustment was carried out using dilute LiOH and HNO_3_. Below pH 3, the potential drift observed is attributed to either the response of SC/Tl^+^-ISEs to H^+^ ions, or it may be due to the protonation of the ionophore itself in the membrane. The observed potential drop above pH 9.5 is attributed to the formation of TlOH precipitate.

**Figure 4:**
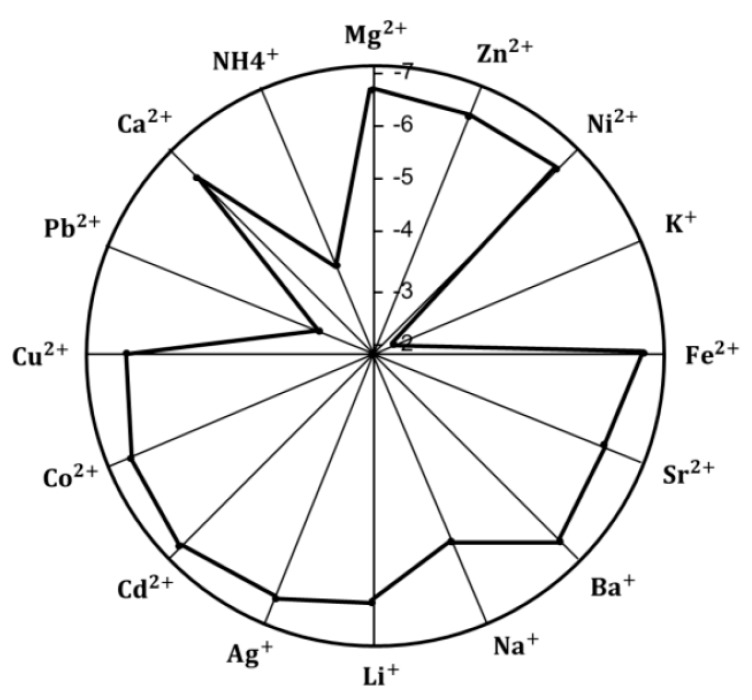
Selectivity coefficient (${\mathrm{Log}\;k}_{\mathrm{Tl},J}^{\mathrm{pot}}$) values of the proposed sensor using modified separate solution method (MSSM).

Potentiometric selectivity coefficients (*log ^Pot^_Tl+, j_*) for the sensor were evaluated using the modified separate solution method (MSSM) [53]. Before measuring the potential response of all tested ions, the membrane had never been in contact with the primary ions. In order of decreasing discrimination, we first started with the most discriminated ions. The highest measured concentrations (10^−1^ M) were used for the estimation of the selectivity values [53]. The activities of both Tl+ and the interfering ions were calculated by the extended Deby–Huckel equation. The results are shown in Figure 4. The selectivity order of the sensor was found to be: Tl^+^> K^+^> Pb^2+^> NH_4_^+^> Li^+^> Cu^2+^≈ Sr^2+^≈ Ca^2+^> Ni^2+^≈ Na^+^> Zn^2+^≈ Ag^+^ ≈ Co^2+^≈ Ba^+^> Mg^2+^≈ Fe^2+^> Cd^2+^. It can be seen that the sensor reveals enhanced selectivity towards Tl^+^ ions over many of the ions under investigation, so the designed sensor is appropriate for the intended estimations.

### 3.2. Formation Constantβ_ML_

The potentiometric selectivity of the polymeric membranes is related to the differences in the lipophilicity of the ions, the membrane composition, and the stability constant [54]. As the last factor is typically the most important one, several methods suitable for the estimation of stability constant values in the membrane phase were proposed [47,48]. Among them, the sandwich membrane method seems to be the most appropriate due to its simplicity and reliability. In this work, various cations and dibenzo-18-crown-6 formation constants were determined. The obtained results are presented in Table 2. It was found that the Tl^+^ ion can form the strongest complex with dibenzo-18-crown-6. The complex stability constants, log *β_ML_*, measured for other cations, are significantly lower compared to thallium, while the values of log *β_ML_* for K^+^ and Pb^2+^ are nearly the same.

### 3.3. Water Layer Test

The water layer, which is formed between the underlying conductor and the ISM, leads to some failures, the most important of which are responsive hysteresis, potential instability, and mechanical problems [19,24]. A simple protocol was presented byPretsch et al. [55] in order to confirm the presence of the water layer between the sensing membrane and the electron conducting substrate. The possible drift by replacing the primary ions solution can be observed with discriminated interfering ions, and the reverse is true in the presence of this layer of water. When interfering ions are replaced by primary ions, or vice versa, either a possible negative or positive potential driftis obtained. This is due to the transfer of the corresponding ions from the samples spread over the ISM, which leads to a change in the composition of the water layer [19,24].

**Figure 5:**
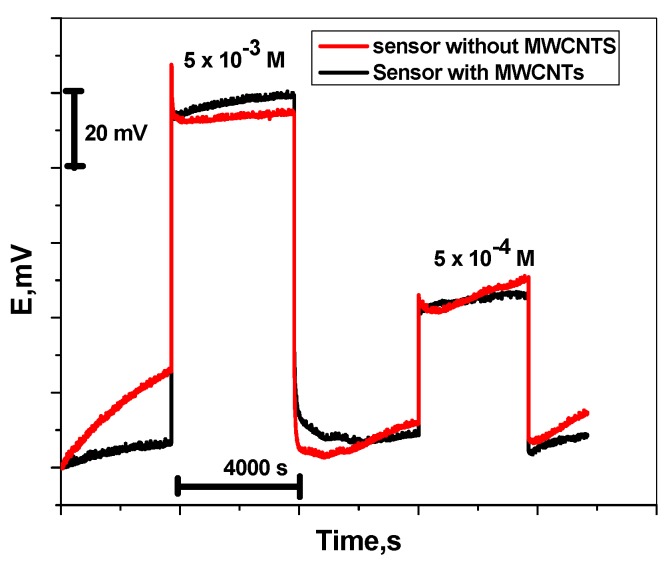
Water layer test for Tl membrane based sensor in the absence and presence of MWCNTs.

Herein, tests for the reduction of the water layer after the insertion of the lipophilic MWCNT layers were carried out. Sequential immersions of the gold plate electrode in the presence and absence of the MWCNT layer in 1.0 × 10^−2^ M Trizma buffer (pH ~ 6) solution was done for the first hour then in a 1.5 × 10^−5^ M Tl (I) solution for the second hour, then in 1.0 × 10^−2^ M Trizma buffer (pH 6) solution for the third hour, then in 2.5 × 10^−6^ M Tl (I) solution, then in 1.0 × 10^−2^ M Trizma buffer (pH ~ 6) solution. The data obtained are illustrated in Figure 5. In the case of the electrode without the MWCNT layer, positive potential drift was observed with a positive potential drift of ~26 mV in 1 h. Subsequently, when the solution was changed to the 1.5 × 10^−5^ M Tl (I) solution, a negative potential drift of 15 mV occurred for the second hour, while using Tl (I) concentration (2.5 × 10^−6^ M) a positive potential drift of 5 mV occurred. Notably, in the case of the presence of the MWCNT layer (GPE/MWCNTs), nearly no significant potential drifts (≈5 mV) were observed when the measured solution was changed from 1.5 × 10^−5^ M to 2.5 × 10^−6^ M. From all of the above results, we can verify that a reduced water layer is formed between the underlying gold plate, the MWCNT layer, and the ISM.This also proves the high hydrophobicity of the intermediate multiwall carbon nano-tube uniform layer.

### 3.4. Chronopotentiometric Test

**Figure 6:**
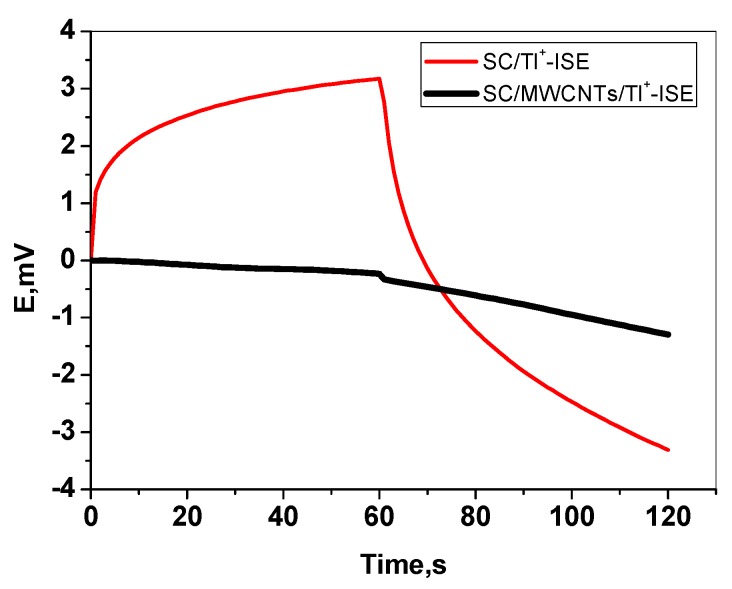
Chronopotentiograms for the solid-contact(SC/Tl^+^-ISE) (top) and SC/MWCNTs/Tl^+^-ISE (bottom) electrodes under the constant currents of ±1 nA in 1.5 × 10^−5^ M of Tl^+^ solution.

At a constant current of ±1 nA, chronopotentiograms were recorded to verify the potential stability of the developed GPE/MWCNTs, and the results are clearly shown in Figure 6. As can be seen in the case of GPE in the absence of MWCNTs, there is a large potential drift of up to 100 ± 0.01 µV/s (*n* = 3). Nevertheless, in the case of GPE/MWCNTs, a much smaller potential drift value of 16 ± 0.02 µV/s (*n* = 3) is recorded for the SC-ISE. Due to the high double layer capacitance of MWCNTs, this is likely to result in potential stability. Using the method provided by Bobacka [56], the membrane resistance and capacitance of the ISE can be calculated. According to the fundamental equations: ∆*E*/∆*t* = *i/C* and ∆*E* = *I* *R*, in which ∆*E*, ∆*t*, *I*, *C*, and *R* represent the change of potential, variation of time, applied current, capacitance, and bulk membrane resistance, respectively, the capacitances in the absence and presence of MWCNTs were calculated to be 23.8 ± 5.1 and 57.8 ± 1.3 µF, respectively. The resistances of the membrane in the absence and presence of the MWCNT layer were 393 ± 9.1 and 100 ± 6.3 kΩ, respectively. From the results shown above, we can confirm the relationship between the potential stability and the presence of the MWCNT layer as a solid contact.

### 3.5. Electrochemical Impedance Spectroscopy (EIS) Measurements

**Figure 7:**
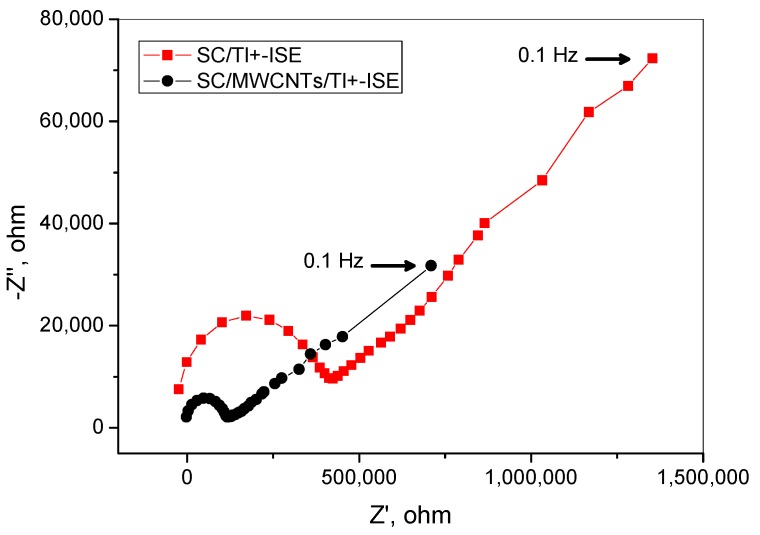
Electrochemical impedance spectroscopy (EIS)spectra of the SC/MWCNTs/Tl^+^-ISE with (circles) and SC/Tl^+^-ISE (squares) without the solid-contact layer of MWCNTs measured in 1.5 × 10^−5^ Tl (I) solution.

The electrochemical impedance spectrum for gold plate electrode (GPE) in the presence and absence of MWCNTs as an ion-to-electron transducer were measured by EIS measurements. The obtained EIS plots are shown in Figure 7. The bulk membrane resistance, together with contact resistance between the underlying conductor and the ISM, is represented by the diameter of the semicircle at the high frequency region, represented by R_bc_, in parallel with the combined geometric capacitance (C_g_) [57]. While at the low-frequency region, characteristics are attributed to the double-layer capacitance (C_d_) coupled with a charge-transfer resistance (R_ct_) at the interface between the ISM and the underlying conductor [57]. After inserting the MWCNT layer, a small R_bc_ can be obtained for the GPE/MWCNTs (R_bc_ = 114.9 ± 12 kΩ) when compared with the GPE in the absence of MWCNTs (R_bc_ = 418 ± 11.2 kΩ). The geometric capacitances (C_g_) for GPE in the absence and presence of MWCNTs are 52.1 ± 3.3 and 141.8 ± 7.4 pF. At the low frequency region, the charge-transfer resistance (R_ct_) and double-layer capacitance (C_dl_) for GPE in the absence and presence of MWCNTs areR_ct_ = 454.5 ± 13.2 kΩ, Cd = 22 ± 2.1 µF and R_ct_ = 200 ± 13.2 kΩ, C_d_ = 50 ± 4.2 µF, respectively. As a result, the presence of MWCNTs as an ion-to-electron transducer offers higher potential stability. This is because of the presence of the MWCNT layers, which facilitate the charge transport between interfaces in the SC-ISEs.

### 3.6. Analytical Application

**Figure 8:**
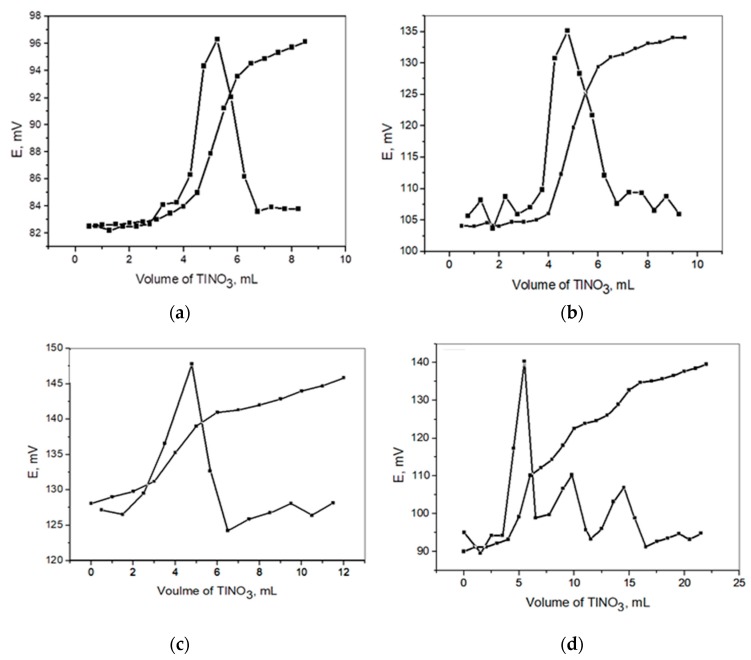
Potentiometric titration curves of NaI (**a**), Na_2_S (**b**), NaBr (**c**), and a mixture of them (**d**) in solution (50 mL) using 0.01 M TINO_3_ titrant (i.e, subfigure represents the 1^st^ derivative for the titration).

The proposed sensor was successfully applied as an indicator electrode in the potentiometric precipitation titration of the Na_2_S, NaBr, and NaI solutions with TlNO_3_. Typical results for the titration of 50.0 mL of 2 × 10^−3^ M S^2−^, 1 × 10^−3^ M Br^−^, and I^−-^ solutions of each of them singly and ternary against 0.01 M TlNO_3_ are shown in Figure 8a–d. It is clear that the concentration of sulfide, bromide, and iodide ions in solution can be accurately determined from the resulting neat titration curve providing a sharp end point.The solubility product of each salt is responsible for the obtianed inflection break, and is the controller for the order and sloping of the potential jump.

## 4. Conclusions

In summary, we presented here for the first time a solid-contact Tl^+^-ISE based on lipophilic multi-walled carbon nanotubes (MWCNTs) as an ion-to-electron transducer over a gold substrate with dibenzo18-crown-6 as a neutral carrier. The optimized formulation of the membrane resulted in a linear concentration range of 4.5 × 10^−6^–7.0 × 10^−4^ M with a slope of 57.30 mV/decade and a limit of detection of 3.2 × 10^−7^ M. The electrode revealed a stable potential over the pH range of 3.0–9.5. The introduction of a MWCNT layer as a high double layer capacitance to the gold plate electrode (GPE), offers the advantages of low electrical resistance and high response stability. The advantages and limitations of many of the previously suggested potentiometric solid-contact thallium sensors are given in Table 3. for comparison. It can be seen that the sensors suggested in the present work have several inherent advantages over many of those previously described. The proposed solid contact electrode is used successfully as an indicator electrode in potentiometric precipitation titrations for singly and ternary mixtures of sulfide, bromide, and iodide anions.

## Figures and Tables

**Table 1:** Potentiometric response characteristics of Tl^+^ PVC (o,NPOE) membrane sensors based ondibenzo-18-crown-6 (DB18C6).

Parameter *	Ionophore	
	DB18C6	DB18C6 + Anionic Additive
Slope, (mV/decade)	32.7 ± 2.5	57.3 ± 1.6
Correlation coefficient, (r)	0.9864	0.9998
Intercept, (mV)	179.5	366.2
Linear range, (M)	7.9 × 10^−6^–7.0 × 10^−4^	4.5 × 10^−6^–7.0 × 10^−4^
Detection limit, (M)	4.0 × 10^−6^	3.2 × 10^−7^
Working range, (pH)	3.0–9.5	3–9.5
Response time for 10^−3^ M, (s)	<10	<10
Accuracy (%)	98.7	99.2
Repeatability (CV_w_, %)	0.9	0.7
Between-day-Variability (CV_b_, %)	1.1	1.2

* Mean of 3 measurements.

**Table 2:** Complex formation constants, Log *β_ML_* of dibenzo 18-crown-6 with various cations, measured in *o*-NPOE plasticized poly (vinyl chloride) membranes.

Ion	Atomic Radius (Å)	Ionophore L_T_ (mmol/Kg)	Additive (mmol/Kg)	Membrane Potential ∆E (mV)	Formation Const.Log β_IL_
Tl^+^	2.2	80	56	258.30 ± 3	5.99 ± 0.6
K^+^	2.8	80	56	252.39 ± 5	5.89 ± 0.8
Pb^2+^	2.02	80	56	255.48 ± 1	5.81 ± 0.7
NH_4_^+^	1.40	80	56	193.00 ± 3	4.88 ± 0.3
Na^+^	2.27	80	56	174.06 ± 1	4.56 ± 0.4

**Table 3:** General characteristics of some potentiometric solid-contact Tl-membrane sensors.

Sensing Material	Transducer	Electrode Material	Stability (Drift)	Slope, mV/Decade	Detection Limit, M	Working pH Range	Selectivity Coefficient (log K^Pot^_Tl__,B_)	Ref.
Calixarene derivatives	(3-octylthiophene)	Au	<0.4 mV/h	58.4	3.02 × 10^−8^	4–9	Zn^2+^(−6.12), Ca^2+^(−6.01), Ba^2+^(−5.84), Cu^2+^(−5.81), Cd^2+^(−5.57), Al^3+^(−5.62), Pb^2+^(−4.52),Li^+^(−3.97), Na^+^(−3.74),H^+^(−3.66), K^+^(−2.77), NH_4_^+^(−2.71), Cs^+^(−2.17) and Ag^+^(−1.16)	[58]
4′-nitrobenzo-18-crown-6	-	Graphite	-	57.2	1.0 × 10^−8^	5–14	K^+^(−0.98), Na^+^(−3.56), Ca^2+^(−1.77) and Mg^2+^(−3.85).	[59]
*N*,*N*′-Dioctylethylenediamine-*N*,*N*′-disuccinic acid	Polyaniline	Pt	-	56 ± 2	8.2 × 10^−8^	4.7–9.0	-	[60]
dibenzo-18-crown-6	Multi-walled carbon nanotubes	Au	16 ± 0.02 µV/s	57.3 ± 1.6	3.2 × 10^−7^	3.0–9.5	Zn^2+^(−6.50), Ca^2+^(−6.35), Ba^2+^(−6.59), Cu^2+^(−6.31), Cd^2+^(−6.7), Pb^2+^(−2.81), Li^+^(−3.97), Na^+^(−6.48), Ni^2+^(−6.48), K^+^(−2.12), NH_4_^+^(−3.48), Mg^2+^(−6.62), Fe^2+^(-6.62), Sr^2+^(−6.35) and Ag^+^(−6.56)	This work
